# Defined host factors support HBV infection in non‐hepatic 293T cells

**DOI:** 10.1111/jcmm.14944

**Published:** 2020-01-12

**Authors:** Xiaoqiang Yang, Weiwen Cai, Xiaoyue Sun, Yanwei Bi, Chui Zeng, XiaoYu Zhao, Qi Zhou, Tian Xu, Qingdong Xie, Pingnan Sun, Xiaoling Zhou

**Affiliations:** ^1^ Stem Cell Research Center Shantou University Medical College Shantou China; ^2^ The Center for Reproductive Medicine Shantou University Medical College Shantou China; ^3^ Guangdong Provincial Key Laboratory of Infectious Diseases and Molecular Immunopathology Shantou University Medical College Shantou China; ^4^ Medical Research Center Sun Yat‐Sen Memorial Hospital Sun Yat‐Sen University Guangzhou China

**Keywords:** HBV, HNF4α, non‐hepatic cells, NTCP, nuclear hormone receptors, PPARα, RXRα

## Abstract

Hepatitis B virus (HBV) is a human hepatotropic virus. However, HBV infection also occurs at extrahepatic sites, but the relevant host factors required for HBV infection in non‐hepatic cells are only partially understood. In this article, a non‐hepatic cell culture model is constructed by exogenous expression of four host genes (NTCP, HNF4α, RXRα and PPARα) in human non‐hepatic 293T cells. This cell culture model supports HBV entry, transcription and replication, as evidenced by the detection of HBV pgRNA, HBV cccDNA, HBsAg, HBeAg, HBcAg and HBVDNA. Our results suggest that the above cellular factors may play a key role in HBV infection of non‐hepatic cells. This model will facilitate the identification of host genes that support extrahepatic HBV infection.

## 1 INTRODUCTION

Hepatitis B is one of the major diseases of mankind and affects the lives of more than 2 billion people. WHO estimates that in 2015, approximately 257 million people were chronically infected with hepatitis B virus (HBV) worldwide, causing about 887 000 deaths per year due to HBV‐associated liver pathologies.^1^ Since drugs for HBV treatment can reduce HBV replication, but rarely result in complete cure, HBV‐related liver diseases remain a major public health problem.^2^, ^3^ As a human hepatotropic virus, HBV mainly replicates in and damages the human liver, but at the same time, HBV infects other cell types in extrahepatic tissues, such as lymph nodes, spleen, kidney, periadrenal ganglia and testis.^4^, ^5^ HBV viral replication in these tissues plays an important role in the pathogenesis of extrahepatic syndromes.^6^ Our research group previously reported the existence of HBV DNA in the chromosomes of sperm isolated from chronic hepatitis B patients. HBV genes can also be expressed in early embryonic cells originating from hamster oocytes fertilized with human spermatozoa transfected with the complete viral genome,^7^, ^8^ and HBV infection can adversely affect human sperm due to HBV S protein‐mediated loss of sperm mitochondrial membrane potential, resulting in reduced sperm motility.^9^ However, we found that the asialoglycoprotein receptor (ASGR) was not expressed in human testis, suggesting that HBV might invade spermatogonial cells through other receptors.^10^ Although such restricted non‐hepatic infection of HBV has been observed, the host factors required for HBV infection in non‐hepatic cells remain only partially understood.

Currently, cell culture models for studying extrahepatic HBV infection are limited. Hepatoma‐based cell culture models, such as HepG2 and Huh7, are widely used in HBV research. However, it is not an easy task to identify key factors in HBV infection from a vast number of liver‐enriched genes expressed in these human hepatoma cells. Reconstitution of HBV infection in non‐hepatic cells will identify host factors that support HBV entry, transcription and replication. Up to now, multiple factors related to HBV infection and replication have been discovered. HBV entry can be mediated by heparan sulphate proteoglycan (HSPG) such as Glypican‐5 (GPC5), sodium taurocholate co‐transporting polypeptide (NTCP).^11^, ^12^, ^13^, ^14^ HBV transcription is also regulated by liver‐enriched transcriptional factors such as hepatocyte nuclear factors (HNF) including HNF1, HNF3 and HNF4, CCAAT/enhancer‐binding protein (C/EBP), and nuclear hormone receptors such as RXRa and PPARa.^11^, ^15^, ^16^, ^17^, ^18^ The initial step of HBV infection involves binding of the virus to a receptor on the target cell surface and initiating viral entry. The preS1 domain of HBV mediates attachment of the virus to its target cell.^19^ HBV particles bind the ASGR in a process that is inhibited by preS1,^20^ indicating ASGR facilitates HBV infection.^21^ In addition, HSPG (eg, GPC5) is required for the first steps of HBV infection.^12^, ^13^, ^22^ Recent reports suggest the sodium taurocholate co‐transporting polypeptide (NTCP) to be a functional HBV cell entry factor. NTCP physically interacts with the N‐terminal domain of preS1 and mediates viral entry into hepatocytes. Most recently, epidermal growth factor receptor has been discovered to play a critical role in mediating HBV‐NTCP internalization into hepatocytes.^23^ With the identification of the receptor, hepatoma cell lines that overexpress NTCP are susceptible to HBV infection.^14^, ^24^ As a bile salt transporter, NTCP is strongly expressed in parenchymal liver cells and is localized to the sinusoidal membrane.^25^ The expression NTCP in human non‐hepatic cells and hepatoma cells is lower than in human primary hepatocytes, which will limit HBV entry into these cells. Moreover, cell lines of non‐hepatic origin do not independently support HBV replication due to a deficiency of some liver‐enriched transcription factors. HBV pregenomic RNA (pgRNA) cannot be detected even when HBV‐containing plasmids are transfected into mouse non‐hepatic NIH3T3 cells.^18^, ^26^ Liver‐enriched transcription factors, such as hepatocyte factor and retinoid X receptor α (RXRα), can bind to HBV promoters and regulate the transcription of HBV genes.^27^ Nuclear hormone receptors (HNF4α, RXRα, PPARα) are essential for pgRNA synthesis and viral replication.^18^, ^27^, ^28^

To reconstitute HBV infection in non‐hepatic cells, we ectopically express multiple genes participating in HBV entry, transcription and replication. In this study, we show that co‐expression of the nuclear hormone receptors HNF4α, RXRα and PPARα, and the HBV receptor NTCP support the entry and replication of HBV in 293T cells. This model will facilitate the study of HBV infection in non‐hepatic cells as well as host factors required for liver tropism of HBV.

## 2 MATERIALS AND METHODS

### 2.1 Plasmid constructs

Plasmids expressing nuclear hormone receptors HNF4α, PPARα and RXRα were obtained from Addgene (FR‐HNF4α, Addgene plasmid 31100; pSG5‐PPARalpha, Addgene plasmid 22751; pSV‐SPORT‐RXRalpha, Addgene plasmid 8882). Plasmid pBR322‐HBV1.0 or pBR322‐HBV2.0 (sub‐type, Adr) were engineered by inserting a 1 or 2 copies of the HBV genome into the cloning vector pBR322, which is kind gift from Professor Yiping Hu of the Second Military Medical University. The plasmid of pcDNA‐HBV1.1 containing 1.1‐copies of the HBV genome from the p3.6 II/HBV, a gift from Professor Wensheng Sun and Professor Xiaohong Liang,^29^ in which HBV genome is located downstream of the CMV promoter in pcDNA3. The pSIN‐NTCP‐EGFP plasmid, expressing a fusion protein of NTCP and EGFP, was constructed by inserting an NTCP‐EGFP fragment into the multiple cloning site of pSIN‐biasic.

### 2.2 Cells and generation of stable cell line

Human hepatoblastoma cells (HepG2) were obtained from the American Type Culture Collection, and human embryonic kidney cells (HEK293T) were provided by Type Culture Collection of the Chinese Academy of Sciences, Shanghai, China. A plasmid encoding NTCP‐EGFP and puromycin resistance genes was transfected into HepG2 cells and HEK293T cells to establish HepG2‐NTCP‐EGFP (HepG2‐NE) cells and 293T‐NTCP‐EGFP (293T‐NE), respectively. Plasmids encoding HNF4α, PPARα and RXRα, and puromycin resistance genes were transfected into 293T‐NE cells to establish 293T‐NE‐3NRs. Selection was achieved with 1 μg/mL puromycin (Gibco). Cells were cultured with DMEM/high glucose (HyClone) supplemented with 10% foetal bovine serum and 100 U/mL penicillin, 100 μg/mL streptomycin, in the presence (HepG2‐NTCP‐EGFP cells) or absence (HepG2, 293T cells) of 0.5 μg/mL puromycin. HepG2.2.15 (a hepatoma HepG2 cell line stably transfected with the whole genome of HBV) (Sai Ku Company) were cultured in DMEM/high glucose (HyClone) supplemented with 10% FBS, 100 U/mL penicillin, 100 μg/mL streptomycin and 380 μg/mL G418. All cells were grown at 37°C under a humidified 5% CO_2_ atmosphere.

### 2.3 Cell transfection

When we co‐transfected HNF4α, PPARα, RXRα and pBR322‐HBV1.0 together into 293T cells in 6‐well plate, 0.5, 0.5, 0.5 and 1.5 μg of each DNA, respectively, were used. Transfections were conducted by using Lipofectamine 3000 reagent according to the manufacturer's instructions (Invitrogen). For cells transfected with pSG5‐PPARalpha and pSV‐SPORT‐RXRalpha, all‐trans retinoic acid (Sigma‐Aldrich) at 1 μmol/L and clofibric acid (Sigma‐Aldrich) at 1 mmol/L final concentrations were added to the medium to activate the PPARα and RXRα nuclear hormone receptors.^18^

### 2.4 Lentivirus preparation

Lentiviral vectors individually encoding the three nuclear hormone receptors (HNF4α, RXRα, and PPARα) and human NTCP were constructed. Then, lentivirus was produced through HEK293T transfection and concentrated by using a Lenti‐X™ Concentrator kit (cat.631231, Clontech).^30^ Titrations were quantified by qPCR to be 5.82 × 10^7^ TU/mL (hNTCP), 3.36 × 10^7^ TU/mL (HNF4α), 6.61 × 10^7^ TU/mL (PPARα), and 9.06 × 10^6^ TU/mL (RXRα).^31^ After that, lentivirus was used to transduce 293T cells, followed by selection with 1.0 μg/mL puromycin.

### 2.5 HBV preparation and infection

HBV particles were concentrated from the culture supernatant of HepG2.2.15. Briefly, the supernatant produced by HepG2.2.15 was collected, centrifuged at 350 *g* for 15 minutes cleared through a 0.45 μm filter to remove cell debris, and then concentrated by using Amicon Ultra‐15 (cat.UFC910008, Millipore). The concentrated virus was recovered from the bottom of the reservoir pocket with 200 μL DMEM and stored as a concentrated HBV stock at −80°C. HBV DNA was extracted, and the copy numbers were quantified by an HBV DNA real‐time PCR Assay kit (Da An Gene).All cells were cultured in 24‐well collagen‐coated plates maintained in DMEM/F‐12(Gibco) medium supplemented with 10% FBS, 4 mmol/L Glutamax, 0.1 mmol/L NEAA, 1 mmol/L sodium pyruvate, 100 U/mL penicillin, 100 μg/mL streptomycin, 1 mg/mL puromycin, 40 ng/mL dexamethasone, 20 μg/mL hydrocortisone, and 5 μg/mL insulin. We inoculated HBV with an indicated genome equivalents (GEq)/cell in medium containing 4% PEG8000 (Sigma‐Aldrich) and 2% DMSO (Sigma‐Aldrich) to the cells (over 90% confluent) for 24 hours at 37°C. After 24 hours infection, we remove the HBV inocula and washed the cells three times with PBS. The medium was changed every 2 days. Supernatant and cells were harvested at the indicated time. For replication inhibition, cells were incubated with 0.2 mg/mL lamivudine (LAM) (GlaxoSmithKline). For entry inhibition, cells were incubated with 5 μmol/L cyclosporine A (CsA) (BBI).

### 2.6 Quantitative PCR analysis of HBV cccDNA

To selectively extract HBV cccDNA, transfected or infected cells were lysed with 200 μL of lysis buffer [50 mmol/L Tris‐HCl, Ph 7.4, 10 mmol/L EDTA, 150 mmol/L NaCl, 1% SDS] at 37°C for 20 minutes, followed by addition of 50 μL of 2.5 mol/L KCl and incubation at 4°C overnight. The lysate was then clarified by centrifugation at 14 000 *g* for 30 minutes at 4°C and extracted with phenol and phenol:chloroform. DNA was precipitated with ethanol in the presence of 5 μg glycogen (Shengong), and precipitated DNA was dissolved in 15 μL TE buffer. The prepared DNA sample was then treated with Nde I restriction endonucleases to linearize pBR322‐HBV1.0 or HBV1.1. These restriction endonucleases cut once in pBR322 or pcDNA, but not in HBV DNA. The digested products were treated with PSAD (plasmid‐safe ATP‐dependent DNase, TAKARA), to hydrolyse linear DNA, following the manufacturer's instructions. Then, the PSAD‐treated DNA was diluted as a template for quantitative PCR analysis of HBV cccDNA according to Bowden's method.^32^

### 2.7 RT‐qPCR

Total RNA was extracted using an RNeasy Mini Kit (Qiagen). About 1000 ng of total RNA was reverse transcribed into cDNA with an RT‐PCR Kit (FSQ‐101, TOYOBO), and qPCR was performed using 2× Power SYBR Green Master Mix (Applied Biosystems) and an ABI 7500 machine, with GAPDH for normalization of input RNA. The RT‐qPCR data were analysed by the ΔΔCT method. Primer sequences are listed in Table 1.

**Table 1:** Primer sequences

Gene	Forward (5′ → 3′)	Reverse (5′ → 3′)
HBV pgRNA	TGTTCAAGCCTCCAAGCT	GGAAAGAAGTCAGAAGGCAA
PPAR	TTTCCCTGTTTGTGGCTGCT	CACAATCCCCTCCTGCAACT
RXRA	AACATTTCCTGCCGCTCGAT	GGGTGCTGATGGGAGAATGC
HNF4A	ACATGGACATGGCCGACTAC	CGTTGAGGTTGGTGCCTTCT
GAPDH	CATGAGAAGTATGACAACAGCCT	AGTCCTTCCACGATACCAAAGT
cccDNA‐Bowden CCC1‐F	GCGGWCTCCCCGTCTGTGCC	
cccDNA‐Bowden DRF1‐F	GTCTGTGCCTTCTCATCTGC	
cccDNA‐Bowden CC2‐R		GTCCATGCCCCAAAGCCACC

### 2.8 Time‐resolved immunofluorometric assay (TRIFA)

Cell supernatants containing HBV particles were filtered through a 0.45 μm filter (Millipore), and then 200 μL of supernatant was heated at 100°C for 15 minutes to inactivate HBV. The quantity of HBV was determined with a EU3+ labelled antibody using a TRIFA kit (Fenghua Bioengineering Company) and detected on a Tecan Infinite 200 Pro plate reader (ex. 340 nm/em. 612 nm).

### 2.9 ELISA

The supernatants were harvested and used to determine the concentrations of HBsAg and HBeAg using ELISA test kits (KeHua) following the manufacturer's instruction. Absorbance was determined with a microtitre plate reader (Quant, Biotek) by dual‐wavelength measurement (450/630 nm).

### 2.10 Immunofluorescence

Infected cells were washed twice with PBS gently, fixed with −20°C methanol for 30 minutes, washed with PBS after removal of the methanol and then blocked with 5% bovine serum albumin (BSA) for 1 hour. Then, fixed cells were incubated with different primary antibodies as follows: anti‐HBc (1:400 dilution, ZSGB‐BIO), anti‐HBs (1:400 dilution, ZSGB‐BIO), anti‐HNF4α (1:800 dilution, Sigma‐Aldrich), anti‐PPARα (1:800 dilution, Thermo Fisher) and anti‐RXRα (1:800 dilution, Sigma‐Aldrich) overnight at 4°C. After being washed with 0.1% PBST buffer (PBS + 0.1% tween 20) three times for 10 minutes each time, cells were incubated with horseradish‐peroxidase‐conjugated goat anti‐rabbit or goat anti‐mouse IgG secondary antibody (PV9000, Zymed Laboratories) for 2 hours at room temperature and then washed with 0.1% PBST buffer another three times. Nuclei were counterstained with 4′6′‐diamidino‐2 phenylindole (DAPI) at room temperature for 5 minutes and finally washed with PBST three times. Fluorescent images were captured using a ZEISS Observer A1 fluorescence microscope.

### 2.11 Western blotting

293T and 293T‐NE‐3NRs cells were washed 3 times with PBS and lysed on ice in RIPA buffer containing 1% protease inhibitor cocktail (Millipore). Protein concentration was determined by a BCA protein assay kit (Beyotime). Protein were electrophoresed on 10% SDS‐PAGE and transferred to a PVDF membrane (Millipore). Then, the membrane was incubated overnight at 4°C with anti‐HNF4α (Sigma‐Aldrich), anti‐PPARα (Thermo Fisher), anti‐RXRα (Sigma‐Aldrich), anti‐SLC10A1 (anti‐NTCP, Sigma‐Aldrich) or anti‐β‐actin (Sigma‐Aldrich), respectively. Proteins were detected by enhanced chemiluminescence (Pierce) and exposed to X‐ray film.

### 2.12 Statistics

All experiments were conducted three times in an independent manner and results are shown as mean ± SD (n = 3). All data are normally distributed, and statistical analyses were performed by GraphPad Prism v5.0 using a two‐tailed Student's *t* test if not specifically noted. For time‐ and dose‐dependent HBV infection experiments, two‐way ANOVA was applied after normality and homogeneity testing. A *P* < .05(*), *P* < .01(**) and *P* < .001(***) were considered as statistically significant. Significant *P* values are indicated by asterisks in the individual figure legends.

## 3 RESULTS

### 3.1 Nuclear hormone receptors activate HBV replication in 293T cells

**Figure 1:**
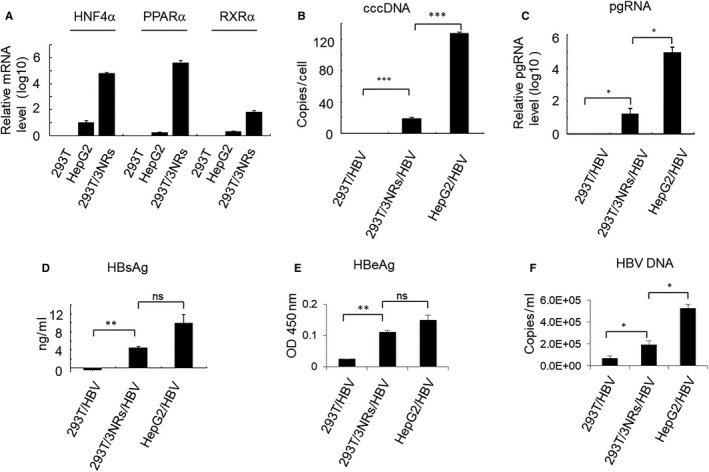
Nuclear hormone receptors activate HBV replication in 293T cells. (A) HNF4α, RXRα, and PPARα expression plasmids were transfected into 293T cells. After 72 h, mRNA expression levels were quantified by qPCR. Control cells were untransfected 293T. ATRA at 1 μmol/L and clofibric acid at 1 mmol/L, were used as ligands to activate the RXRα and PPARα nuclear hormone receptors, respectively. Plasmid pBR322‐HBV1.0 (0.5 μg) was transfected into cells together with or without nuclear hormone receptors (HNF4α 0.2 μg, RXRα 0.2 μg, PPARα 0.2 μg). (B) At 72 h after transfection, HBV cccDNA was quantified by absolute quantitative PCR, and then calculated as copies per cell. (C) HBV pgRNA was detected by RT‐qPCR. (D) HBsAg was detected by time‐resolved fluorescence. (E) HBeAg was detected by ELISA. (F) HBV DNA in the supernatant was extracted and quantified using an HBV DNA real‐time PCR Assay kit. Results are the means ± SD of three repeats, with differences assessed using Student's *t* test (*P < .05, **P < .01, ***P < .001)

To improve HBV replication, expression plasmids encoding the nuclear hormone receptors (HNF4α, PPARα and RXRα) were co‐transfected into 293T cells. Seventy‐two hours post‐transfection, the mRNA fold change level compared with untransfected 293T cells was quantified by qPCR. The mRNA expression level of HNF4α, PPARα and RXRα significantly increased after transfection (Figure 1A).

HBV cccDNA is converted from HBV rcDNA and used as the template transcribed to generate the 3.5 (pgRNA), 2.4, 2.1 and 0.7 kb HBV RNAs. To examine HBV replication in 293T cells, we transfected with HNF4α, PPARα and RXRα, along with pBR322‐HBV1.0 and examined the cells 72 hours after transfection for the presence of HBV cccDNA, HBV pgRNA, HBsAg, HBeAg and HBV DNA. The specific amplification of HBV cccDNA from HepG2 cells (transfected with pBR322‐HBV1.0) and 293T cells (transfected with pBR322‐HBV1.0 or transfected with pBR322‐HBV1.0, HNF4α, PPARα and RXRα) was conducted using the plasmid‐safe ATP‐dependent DNase (PSAD) protocol and primers targeting the rcDNA gap (Figure 1B). As expected, HBV pgRNA was found in nuclear hormone receptor‐transfected 293T cells, indicating that the transfected genes could support HBV replication in 293T cells (Figure 1C). Furthermore, expression of HBsAg, HBeAg and HBV DNA was examined and confirmed to be released into the supernatant from 293T cells transfected with pBR322‐HBV1.0 and three nuclear hormone receptors (Figure 1D‐F). These results indicate that new HBV proteins and DNA were also synthesized followed by the expression of HBV pgRNA. Treatment of 293T cells (transfected with HBV1.0/HBV2.0 and HNF4a, PPARa and RXRa) with the nucleoside analog‐adefovir dipivoxil, to inhibit HBV replication, also reduced HBV pgRNA and HBsAg (Figure S1).

### 3.2 NTCP‐EGFP mediates the entry of HBV into HepG2‐NTCP‐EGFP cells

**Figure 2:**
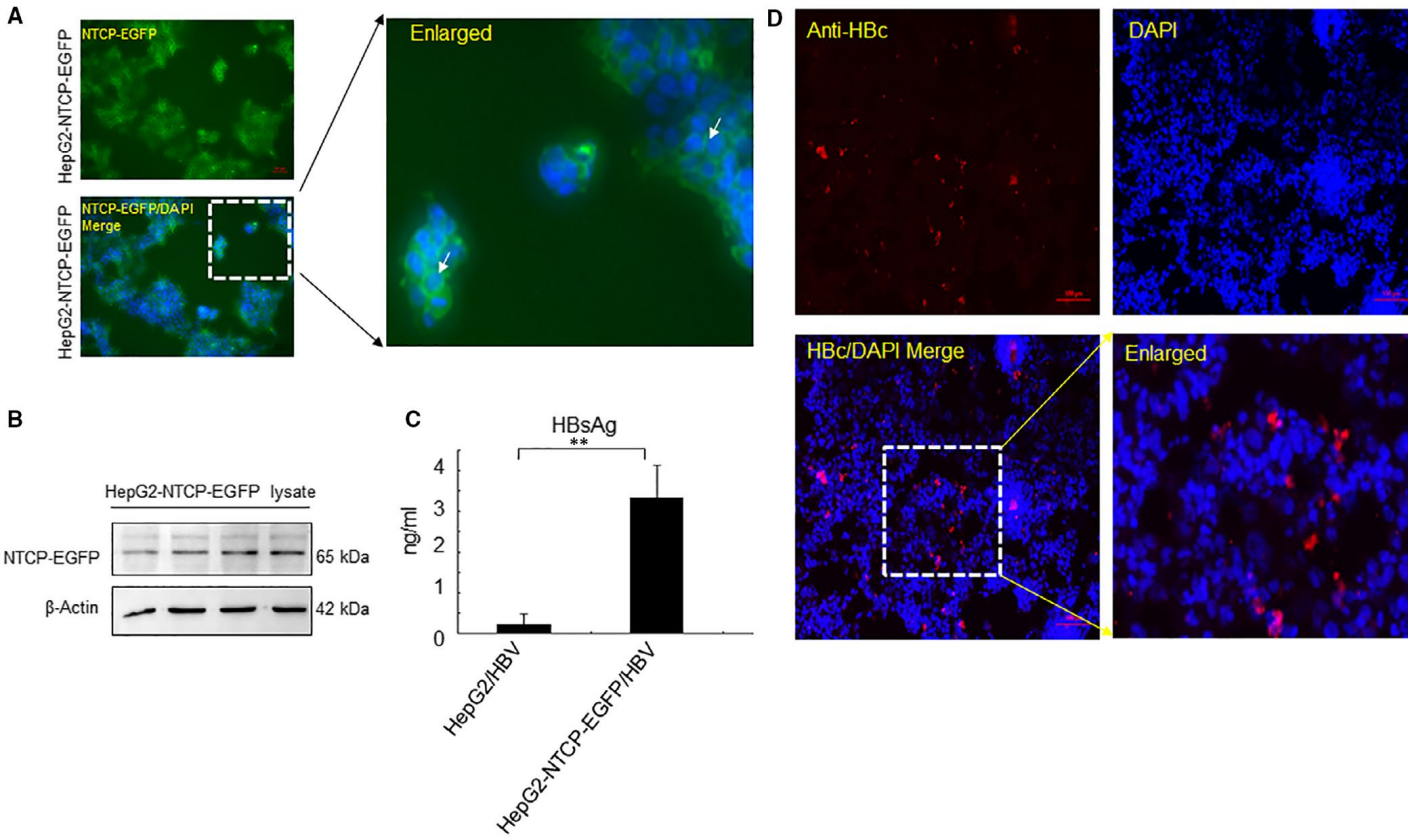
NTCP‐EGFP mediates infection by HBV in HepG2 cells. (A) HepG2‐NTCP‐EGFP cell lines were observed under the fluorescence microscope. The white arrow indicates the EGFP signal on the membrane. (B) Total protein was isolated from HepG2‐NTCP‐EGFP cells, and the expression of NTCP was identified by Western blot (C) HepG2 cells or HepG2‐NTCP‐EGFP cells were infected with HBV (150 Geq/cell) for 24 h. Cells were fixed and supernatant was collected at day 12 d.p.i. Secreted HBsAg was detected by TRIFA. (D) HBcAg expression was determined by indirect immunofluorescence. (Results are the means ± SD of three repeats, with differences assessed using Student's *t* test. **P < .01. Scale bar = 100 μm)

Recently, it was found that susceptibility to HBV infection is determined by NTCP, a functional receptor for HBV entry.^14^ As a multiple transmembrane transporter, NTCP is predominantly expressed at the basolateral membrane of hepatocytes, and exogenous NTCP expression renders non‐susceptible hepatic cells susceptible to HBV infections. To establish a stable and trackable cell line expressing NTCP, we transfected pcDNA3.0‐NTCP‐EGFP into HepG2 cells and selected with 1 μg/mL puromycin. Under a fluorescence microscope, the selected HepG2‐NTCP‐EGFP cells displayed an EGFP signal located in the plasma membrane (Figure 2A). The expression of NTCP in HepG2‐NTCP‐EGFP cells was also confirmed by Western blot (Figure 2B). HepG2‐NTCP‐EGFP cells were inoculated with HBV inoculum overnight and washed in PBS. Fresh culture medium was changed every 2 days. Since HBsAg release from the HBV inoculum was transient and limited, the secreted HBsAg detected 4 days after infection reflected the viral replication level.^33^, ^34^ Here we used the TRIFA method to quantify HBsAg protein. The value of R square was 0.9936, indicating the standard curve was accurate. As expected, HepG2‐NTCP‐EGFP cells produced HBsAg after inoculation of virus, whereas HepG2 cells did not produced HBsAg (Figure 2C). Furthermore, HepG2‐NTCP‐EGFP cells supported productive infection based on immunostaining for HBV core (HBc) protein (Figure 2D). These results suggest that ectopic expression of NTCP‐EGFP in HepG2 cells also enables cells to be infected by HBV.

### 3.3 Productive HBV infection of 293T cells requires expression of NTCP, HNF4α, RXRα and PPARα

**Figure 3:**
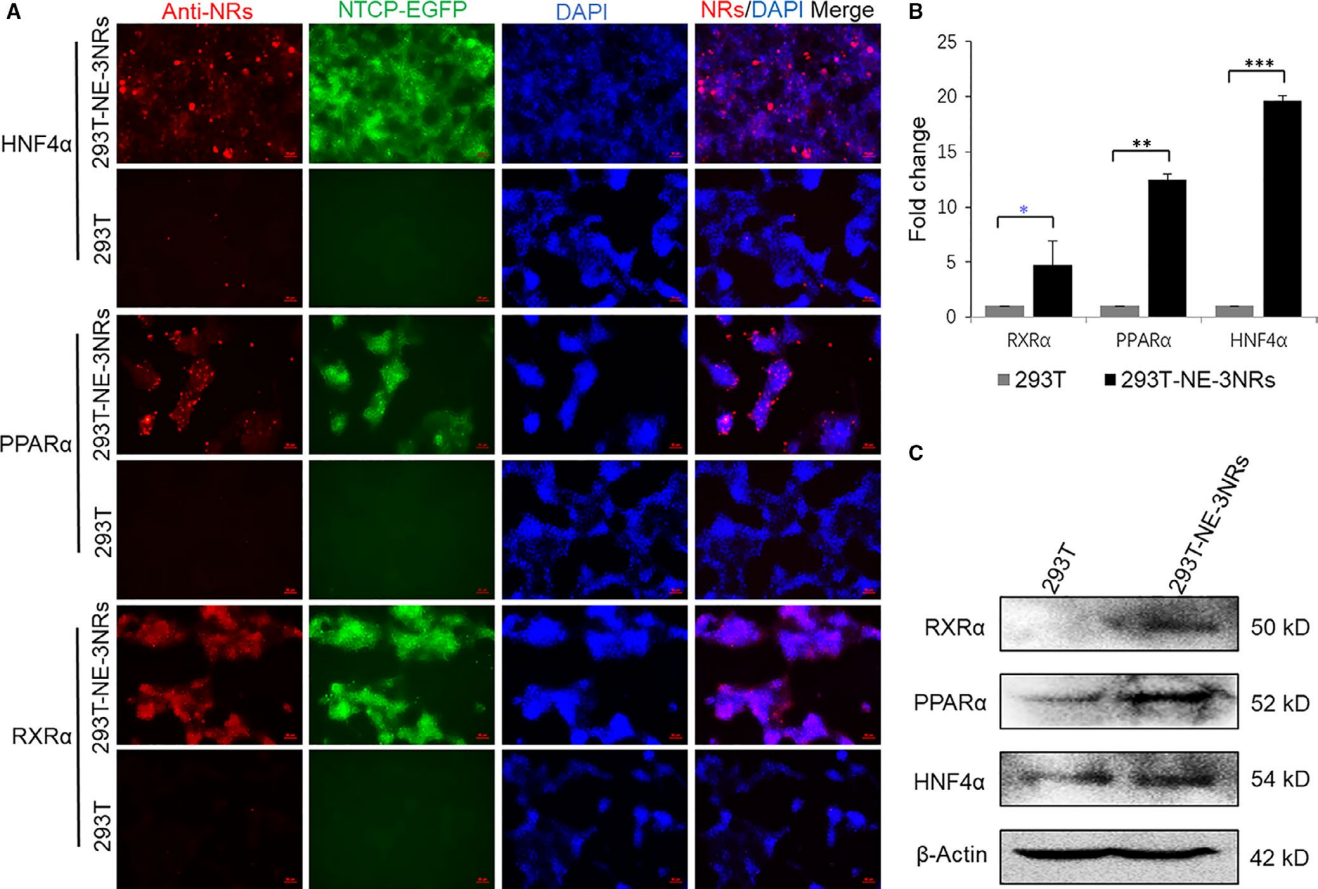
293T‐NE‐3NRs stably overexpressing human NTCP and three nuclear hormone receptors. Lentiviruses individually encoding NTCP and three nuclear hormone receptors were prepared and transduced into 293T cells. (A) Protein expression of HNF4α, PPARα and RXRα were identified by indirect immunofluorescence. 293T cells were used as the control. (B) Total cellular RNA was isolated from 293T‐NE‐3NRs and 293T‐NE, and the mRNA expression level of HNF4α, PPARα and RXRα was quantified by real‐time PCR and normalized to GAPDH. (C) Total protein was isolated from both 293T and 293T‐NE‐3NRs, and the expression of HNF4α, PPARα and RXRα was identified by Western blot. β‐Actin was used as the loading control. (Results are the means ± SD of three repeats. Scale bar = 50 μm, *P < .05, **P < .01, ***P < .001)

As we showed above (Figures 1 and 2), overexpression of the necessary nuclear hormone receptors supported HBV replication in non‐hepatic cells (293T), and human NTCP mediates HBV infection in hepatic cells (HepG2). Therefore, we proposed to develop a new non‐hepatic cell model by overexpressing both the key nuclear hormone receptors and human NTCP. To achieve this, we transduced HNF4α, RXRα, PPARα and human NTCP into 293T cells to generate 293T‐NE‐3NRs (expressing human NTCP, HNF4α, RXRα and PPARα). All encoded receptors were highly expressed in 293T‐NE‐3NRs, based on quantification, immunostaining and Western blot for HNF4α, RXRα and PPARα mRNA or proteins, and fluorescence for NTCP‐EGFP (Figure 3A‐C).

**Figure 4:**
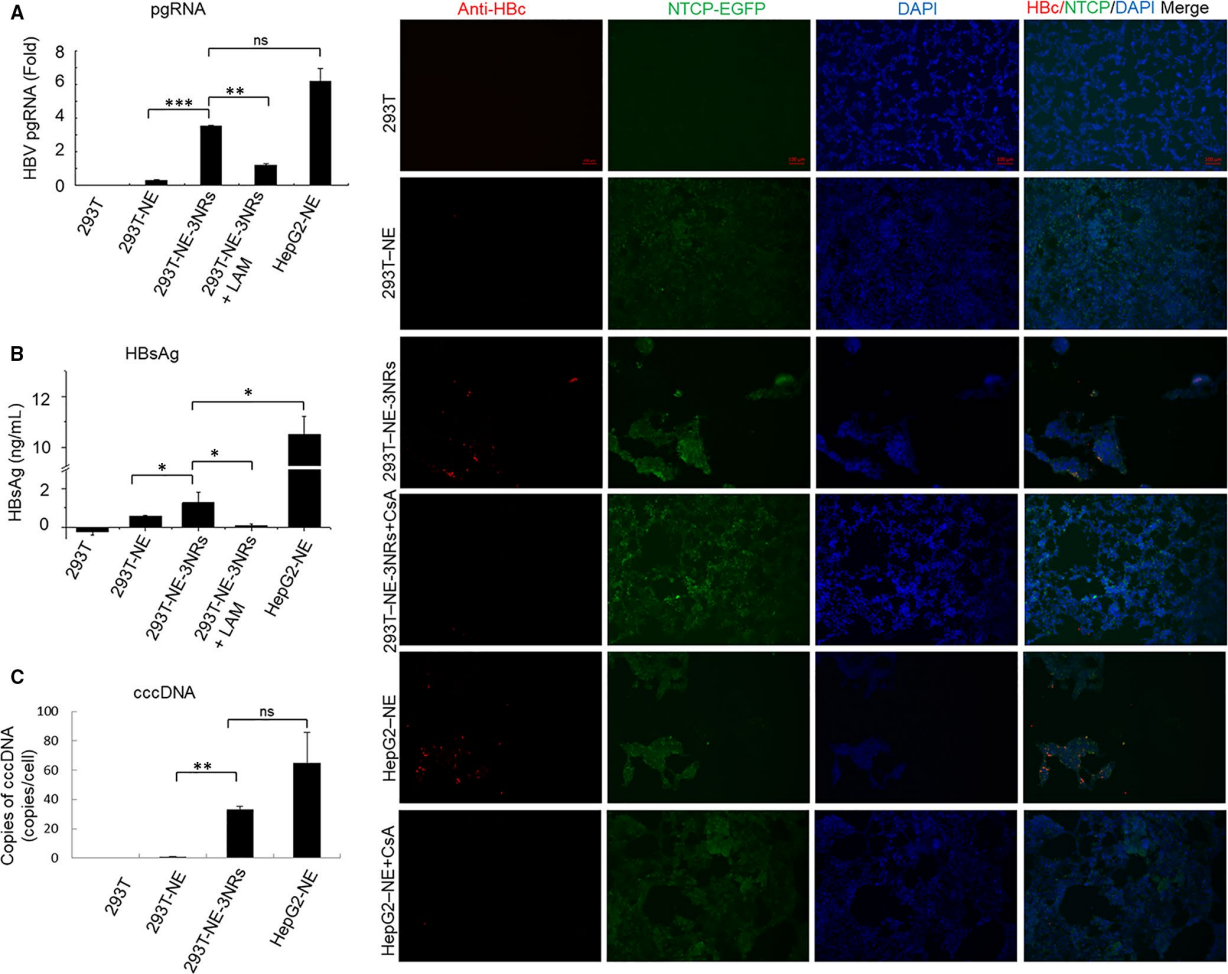
293T‐NE‐3NRs cells support HBV infection and replication. HBV derived from HepG2.2.15 was used to infect 293T, 293T‐NE, 293T‐NE‐3NRs and HepG2‐NE cells with 150 Geq per cell. Total cellular RNA, cccDNA and supernatant were harvested and cells were fixed in −20°C methanol at day 10 d.p.i. (A) HBV pgRNA expression levels were quantified by real‐time PCR. (B) HBsAg was detected by time‐resolved fluorescence assay. (C) HBV cccDNA was quantified by real‐time PCR. Cells in (A, B, C) *P* < .05 293T‐NE‐3NRs vs 293T‐NE (D) HBcAg expression was identified by indirect immunofluorescence. Scale bar = 100 μm; Results are the means ± SD of three repeats, with differences assessed using Student's *t* test (*P < .05, **P < .01,***P < .001). CsA, cyclosporine A

The cells were then infected with HBV and the generation of HBV intermediate products was examined. HBV pgRNA and HBsAg were detected by qPCR and TRFIA, respectively. 293T‐NE‐3NRs cells produced HBV pgRNA after infection in a manner sensitive to inhibition by LAM (Figure 4A). The expression level of HBsAg in 293T‐NE‐3NRs cells was 1.3 ng/mL, higher than the 0.08 ng/mL level observed in LAM‐treated 293T‐NE‐3NRs cells (Figure 4B), suggesting that HBV infected and replicated in the 293T‐NE‐3NRs cells. HBV cccDNA was detected in 293T‐NE‐3NRs cells at a level of 33.3 ± 2.0 copies/cell, whereas not detected in 293T‐NE cells, indicating 293T cells did not support HBV infection when only NTCP was expressed. If NTCP was co‐expressed with the three nuclear hormone receptors (HNF4α, RXRα and PPARα) in 293T‐NE‐3NRs cells, HBV infection could occur and HBV cccDNA could form. As a positive control, infected HepG2‐NTCP‐EGFP cells produced HBV cccDNA at a concentration of 65.1 ± 20.5 copies/cell (Figure 4C).

**Figure 5:**
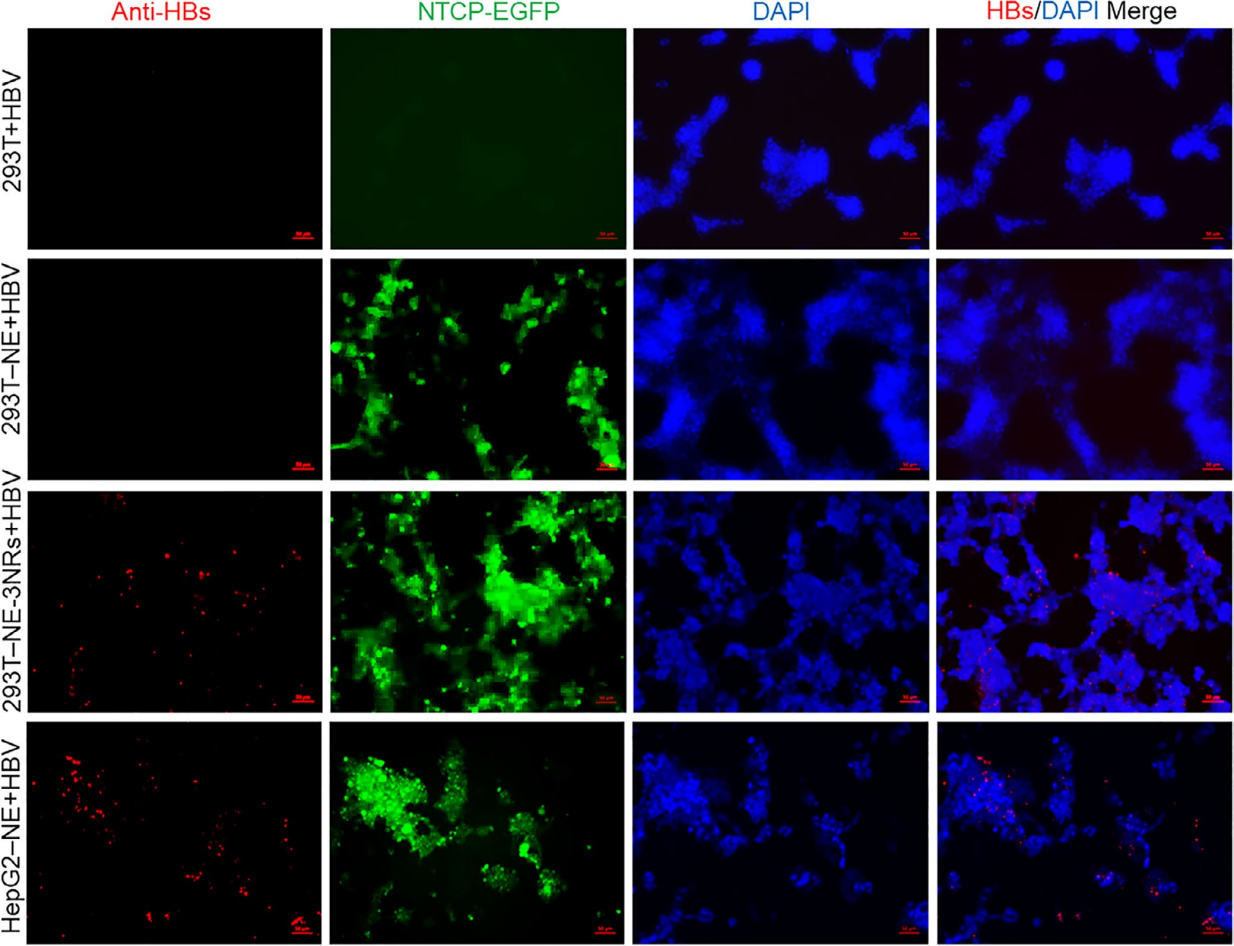
HBsAg expression was identified by indirect immunofluorescence. HBV derived from HepG2.2.15 was used to infect 293T, 293T‐NE, 293T‐NE‐3NRs and HepG2‐NE cells at 150 Geq per cell. Cells were fixed by ice methanol at day 10 d.p.i. Scale bar = 50 μm

Immunofluorescence assay of HBc proteins showed that HBcAg was expressed in 293T‐NE‐3NRs and HepG2‐NE cells, but signals for HBc in 293T‐NE‐3NRs were lower than that in HepG2‐NE. This indicates there may be other important factors that affect HBV infection in HepG2 cells, but are limited in 293T cells. On the other hand, neither 293T nor 293T‐NE expressed HBc, and CsA effectively inhibited HBV entry (Figure 4D). Similar to HBcAg, HBsAg was expressed in 293T‐NE‐3NRs but not in 293T and 293T‐NE cells (Figure 5). We also conducted a time‐ and dose‐dependent HBV infection. For this, two different MOIs (Geq per cell of 100 and 600) were used to infect 293T‐NE‐3NRs cells, whereas 293T‐NE‐3NRs cells treated without HBV and with DMSO were used as a negative control. The infected cells grew slowly after infection (Figure S2). The cell culture supernatants were harvested every 2 days from 5 to 13 d.p.i. Then, HBsAg and HBeAg in the supernatants were determined by TRFIA. Two‐way ANOVA showed a significant dose effect for HBeAg and HBV DNA (*P* < .05), and significant time‐dependent effect was found for HBeAg and HBV DNA between the group infected with 100 Geq per cell and the group infected with 600 Geq per cell (*P* < .05) (Figure S3).

### 3.4 HBV infection occurs in the modified 293T cells co‐cultured with HepG2.2.15

**Figure 6:**
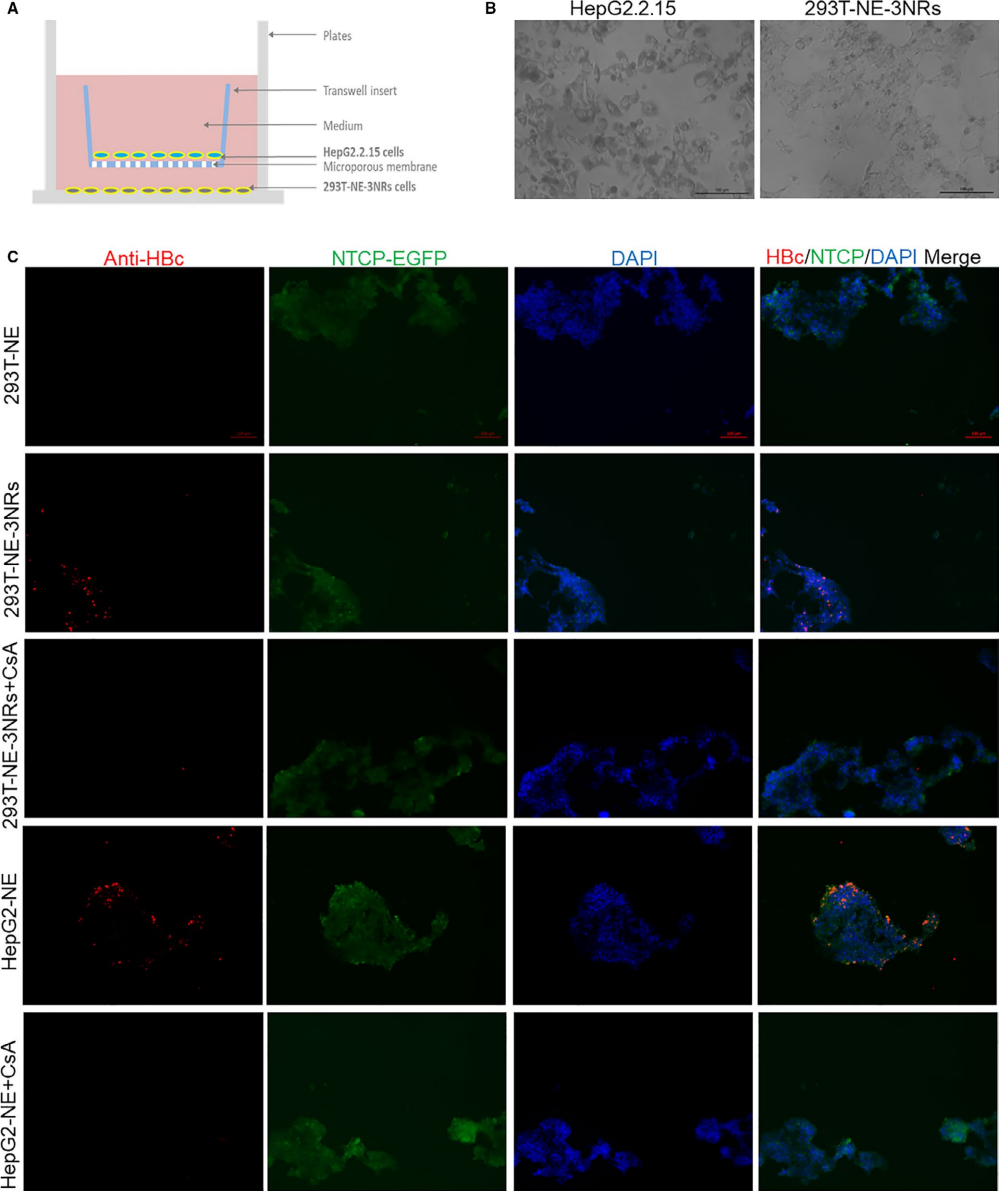
293T‐NE‐3TFs cells are susceptible to HBV infection when co‐cultured with HepG2.2.15 cells. (A) Schematic diagram of the co‐culture 293T‐NE‐3NRs and HepG2.2.15. (B) Cell morphology was observed by microscopy, scale bar = 100 μm. (C) Cells were fixed in −20°C methanol at 10 d post co‐culture. Then, HBcAg was determined by immunofluorescence. CsA, cyclosporine A

In the above HBV infection at high GEq/cell (150), DMSO and PEG8000 were used to increase HBV infection efficiency, and the virus was removed 24 hours after infection. We wondered whether 293T‐NE‐3NRs cells were susceptible to HBV under more natural conditions (at low GEq/cell (about 1.83) and without DMSO and PEG8000). To confirm that modified 293T cell overexpressing hNTCP, HNF4α, RXRα and PPARα could support HBV infection and replication under more natural condition, we co‐cultured 293T‐NE‐3NRs cells or 293T‐NE cells with HepG2.2.15 by using transwells. 293T‐NE‐3NRs cells or 293T‐NE cells were coated in the bottom of the cell culture plate and HepG2.2.15 cells were cultured in the bottom of transwell insert. Ten days after co‐culture, the expression of HBc in 293T‐NE‐3NRs cells could be observed, indicating that 293T‐NE‐3NRs cells can be infected by HBV present in the HepG2.2.15 supernatant (Figure 6). The results indicated that expression of all four genes can confer susceptibility to HBV infection in 293T cells under more physiological conditions.

## 4 DISCUSSION

Previously, conferral of HBV infection to different non‐hepatic human cells had not been successful following transduction of NTCP only. Only human cell lines of hepatic origin reconstituted with hNTCP become HBV susceptible, and HBV replication was still restricted in NTCP‐ overexpressing non‐hepatic human HeLa cells and mouse hepatoma cell lines.^35^, ^36^ In addition, human lung epithelial cells (A549) overexpressing NTCP also do not support HBV infection.^37^ These results suggested that NTCP expression alone is not sufficient to confer HBV infection and that additional liver‐specific factors are required.

In this study, we reconstituted HBV infection in human embryonic 293T cells by ectopic co‐expression of the nuclear hormone receptors HNF4α, RXRα and PPARα, and the HBV receptor NTCP. The first barrier to HBV infection in non‐hepatic cells is a lack of a hepatocyte‐specific virus receptor, now known as NTCP.^14^ The second level of restriction in non‐hepatic cells involves liver‐enriched transcription factors capable of transcribing the HBV genome. Non‐hepatic cells provide a different perspective on the study of HBV infection, especially on the factors responsible for HBV pgRNA synthesis. The expression of this 3.5 kb transcript covering the whole HBV genome is a critical determinant of HBV production.^38^ When HBV pgRNA synthesis is controlled by HBV itself, replication is found only in hepatic cell lines.^18^, ^26^ In the replication of HBV life cycle, expression of the 3.5 kb HBV pgRNA is directed by the HBV nucleocapsid promoter. The transcription factors binding to the HBV nucleocapsid prompter include Sp1,^14^ C/EBP,^33^ HNF3^34^ and HNF4.^38^ More importantly, HNF4α, RXRα and PPARα support HBV replication in murine NIH3T3 fibroblasts by controlling HBV pgRNA synthesis.^18^ Liver‐enriched nuclear receptors (NRs) play a central role in transcribing HBV pgRNA by binding to both HBV enhancer I and HBV enhancer II.^38^ Our results suggest NTCP also mediate the entry of HBV into non‐hepatic cells, and nuclear hormone receptors are among the major positive modulators of viral replication in liver cells. This confirms that human hepatotropic infection of HBV occurs at two distinct levels including viral entry and viral biosynthesis.

However, HBV replication in our engineered 293T cells is still lower than that in HepG2 cells, which may be attributed to two characteristics of 293T cells. First, 293T cells were derived from primary human embryonic kidney and have lower expression of liver‐enriched genes compared with that in hepatic cells such as HepG2 and Huh 7. Even though the expression levels of HNF4α, RXRα and PPARα in the engineered 293T are higher than that in HepG2 cells, HBV production in 293T cells still remains lower than that in HepG2 cells, suggesting that other host factors are also required for robust viral production. Additional relevant factors modulating viral production may be other hepatocyte nuclear factors, such as HNF1 and HNF3,^38^ or miRNAs such as mir‐122^39^ or mir‐372/373.^40^ Different modes of ligand‐mediated activation of HBV transcription and replication have been discovered by utilizing 293T cells.^41^ On the other hand, this 293T cell model involving a non‐hepatic background is suitable for discriminating which are the key hepatic factors from a large number of liver‐enriched genes and discovering other new factors that play a critical role in HBV infection. Second, the innate immune response of this 293T cell model is intact compared with that of hepatoma cells.^42^ This characteristic may also contribute to low level of HBV replication in this model. On the other hand, it could help to discover the restrictive factors for HBV infection in kidney‐derived cells.

In summary, we have engineered human embryonic kidney cells (293T), competent for replicating HBV, by expressing liver‐enriched genes necessary for HBV entry and transcription. We demonstrate that susceptibility to HBV can be conferred to non‐hepatic cells by host cell expression of NTCP, HNF4α, RXRα and PPARα, even though at a very low amounts of HBV. Our engineered 293T cell model provides an alternative way to study HBV infection in other non‐hepatic cells. For example, when investigating HBV infection in spermatogonial cells, we can first examine the distribution of NTCP, HNF4α, RXRα and PPARα in human testis by multiple immunoenzyme histochemistry staining.^10^ Also, further application of this model will help to advance our knowledge of the hepatotropism of HBV and elucidate host and restriction factors responsible.

## CONFLICTS OF INTEREST

The authors declare no conflict of interest.

## AUTHOR CONTRIBUTIONS

XLZ and PNS designed the experiments. XQY, WWC, XYS, PNS, YWB, CZ, XYZ, QZ and TX performed the experiments. PNS, XLZ and XQY analysed data. QDX contributed materials/analysis tools. PNS, XLZ, XQY and WWC wrote the paper.

## Data Availability

The data that support the findings of this study are available in the supplementary material of this article.
