# Supplementary figures and images for: Defined host factors support HBV infection in non‐hepatic 293T cells
Source: J Cell Mol Med. 2020 Jan 12;24(4):2507–18. doi: 10.1111/jcmm.14944 (PMC7028854; doi:10.1111/jcmm.14944)

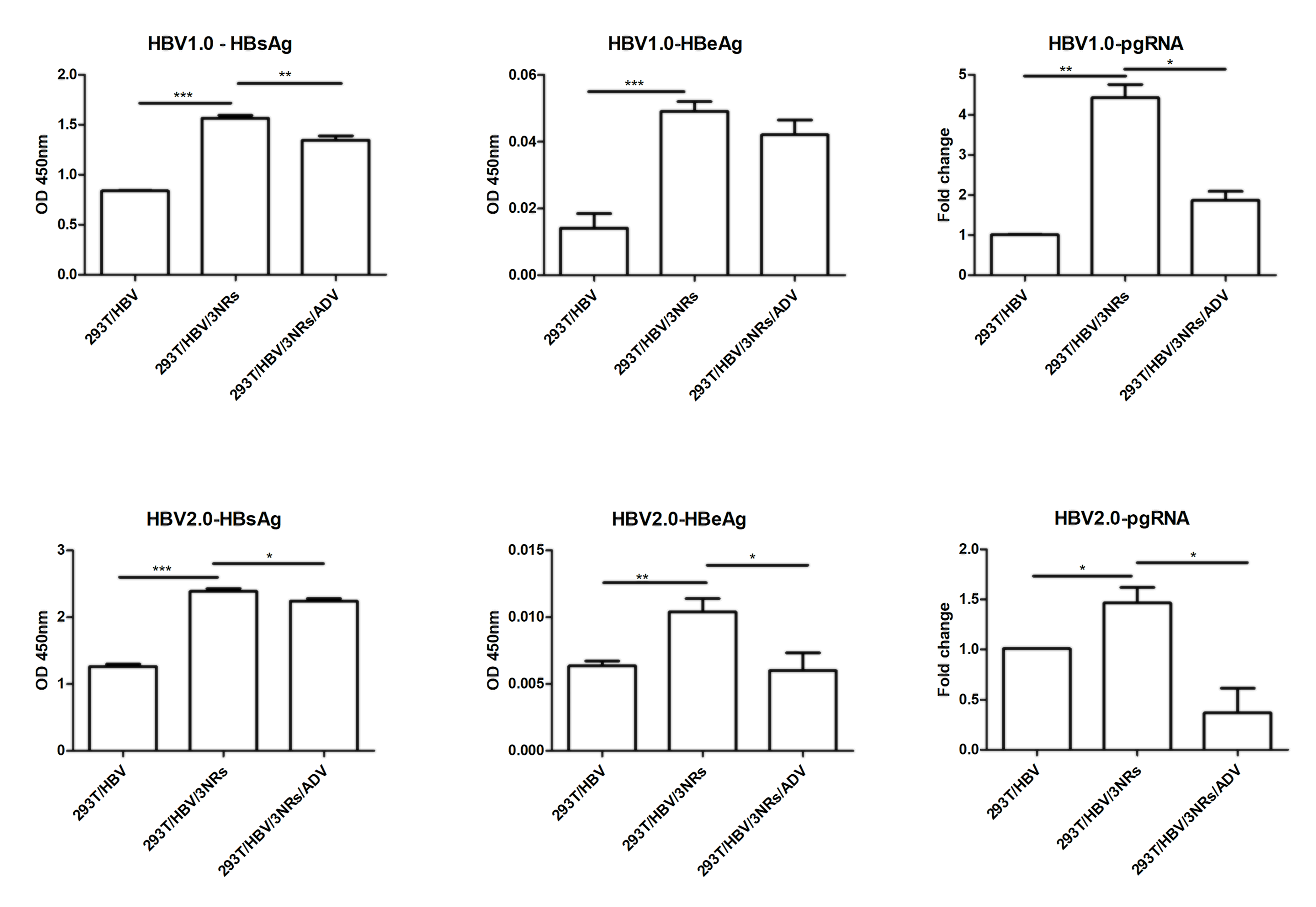

Supplement: Supplementary file 1 [file JCMM-24-2507-s001.tif]

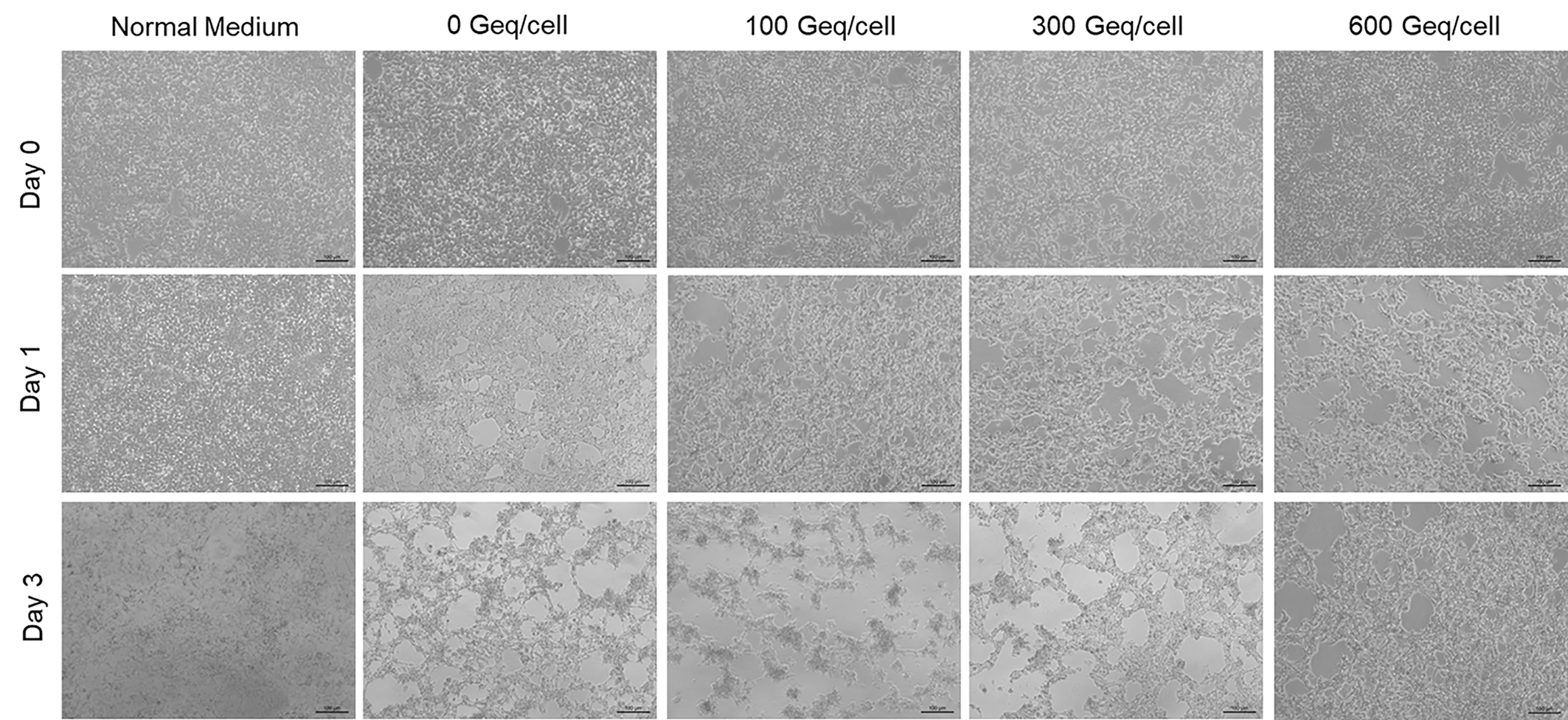

Supplement: Supplementary file 2 [file JCMM-24-2507-s002.tif]

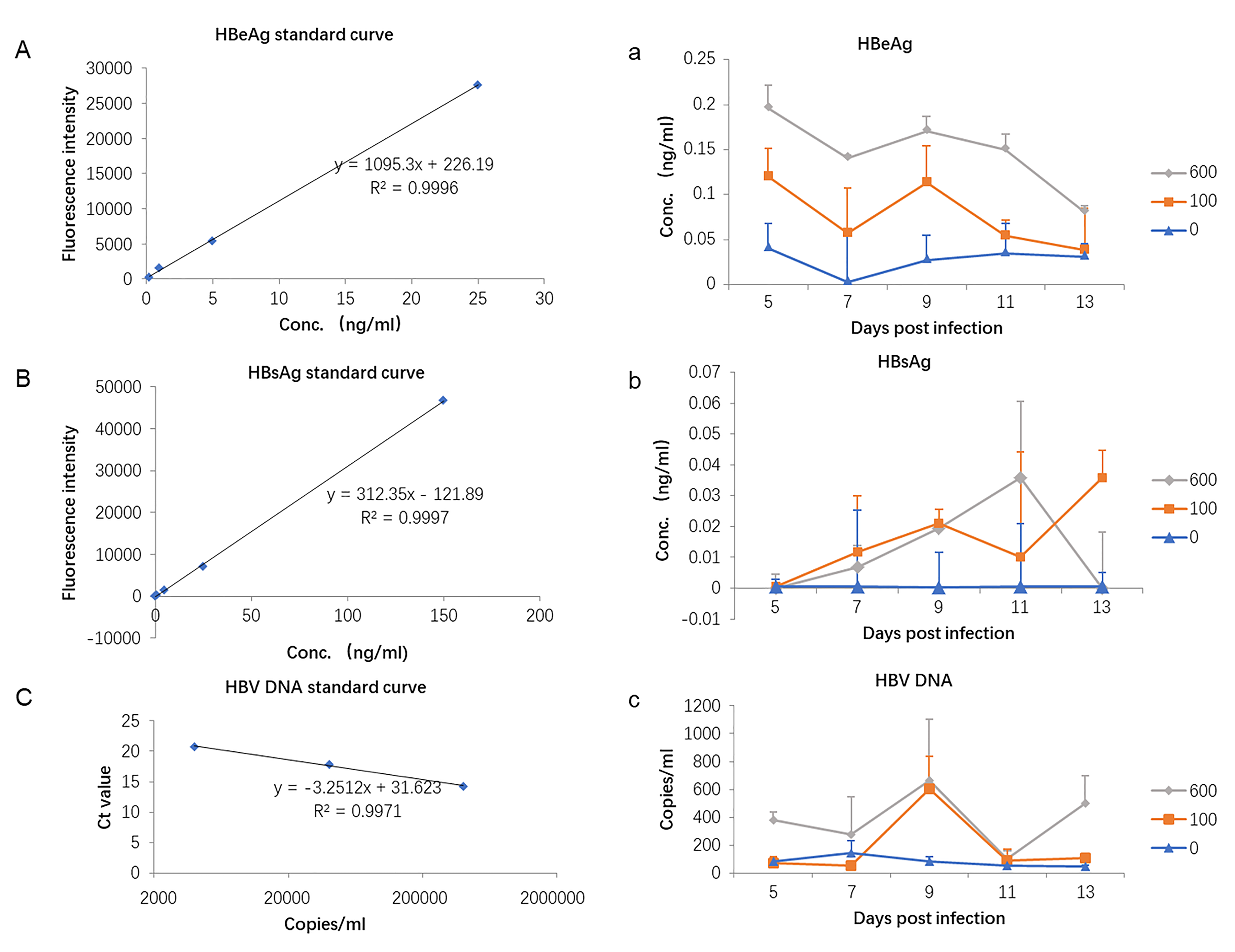

Supplement: Supplementary file 3 [file JCMM-24-2507-s003.tif]
