# Supplementary material for: Defined host factors support HBV infection in non‐hepatic 293T cells
Source: J Cell Mol Med. 2020 Jan 12;24(4):2507–18. doi: 10.1111/jcmm.14944 (PMC7028854; doi:10.1111/jcmm.14944)
Supplement: Supplementary file 4 [file JCMM-24-2507-s004.docx]

**Figure S1** Nuclear hormone receptors support plasmid HBV replication in 293T cells. 293T cells were seeded into 12-well plates and co-transfected with the HBV genome and three nuclear hormone receptors when cells were 50% confluent. The approved anti-HBV drug adefovir (ADV, 1.5 nM) was used to inhibit virus replication. 293T cells transfected with pcDNA3 was set as the negative control. At 72 hours post-transfection, supernatants were collected for detection of HBsAg and HBeAg by ELISA. Total RNA was isolated for pgRNA detection. (A) pBR322-HBV1.0 (B) pBR322-HBV2.0. Data were analysed by Student’s *t* test. (* *P* < 0.05, ** *P* < 0.01, *** *P* < 0.01.)

**Figure S2** Cell growth after HBV infection. 293T-NE-3NRs cells were seeded into 48-well plates and cultured to 95% confluence, then infected with HBV (0, 100, 300, or 600 Geq/cell). 2% DMSO and 4% PEG8000 were used in all groups except normal medium group (DMEM 89%, FBS 10%, P/S 1%). At 24 h after infection, the cells were washed twice with PBS, refed with growth medium and allowed to grow. The total number of cells in the DMSO plus PEG8000-treated cultures was decreased, indicating DMSO and PEG8000 used in the infection process affected cell growth. Scale bar = 100 μm.

**Figure S3** Kinetics of HBsAg, HBeAg and HBV DNA expression in 293T-NE-3NRs cells infected with HBV. 293T-NE-3NRs cells were infected with HBV particles, at two different MOI (Geq per cell of 100 and 600), for 13 days, and supernatant was collected at 5, 7, 9, 11, and 13 d.p.i., followed by fixation of cells at 13 d.p.i.. Then, (a) HBeAg and (b) HBsAg in the collected supernatant was detected by time-resolved fluorescence assay; (c) HBV DNA in the supernatant was isolated with an HBV DNA real-time PCR Assay kit and quantified by real-time PCR. (A-C) are standard curves. Scale bar = 100 μm; Results are the means ± SD of three repeats.
